# Differential expression and role of p21^cip/waf1 ^and p27^kip1 ^in TNF-α-induced inhibition of proliferation in human glioma cells

**DOI:** 10.1186/1476-4598-6-42

**Published:** 2007-06-12

**Authors:** Pabbisetty Sudheer Kumar, Anjali Shiras, Gowry Das, Jayashree C Jagtap, Vandna Prasad, Padma Shastry

**Affiliations:** 1 National Centre for Cell Science, NCCS, Ganeshkhind, Pune 411 007, India

## Abstract

**Background:**

The role of TNF-α in affecting the fate of tumors is controversial, while some studies have reported apoptotic or necrotic effects of TNF-α, others provide evidence that endogenous TNF-α promotes growth and development of tumors. Understanding the mechanism(s) of TNF-α mediated growth arrest will be important in unraveling the contribution of tissue associated macrophages in tumor resistance. The aim of this study was to investigate the role of Cyclin Dependent Kinase Inhibitors (CDKI) – p21^cip/waf1 ^and p27^kip1 ^in TNF-α mediated responses in context with p53 and activation of NF-κB and Akt pathways. The study was done with human glioma cell lines -LN-18 and LN-229 cells, using monolayer cultures and Multicellular Spheroids (MCS) as *in vitro *models.

**Results:**

TNF-α induced inhibition of proliferation and enhanced the expression of p21^cip/waf1 ^and p27^kip1 ^in LN-18 cells. p21 was induced on exposure to TNF-α, localized exclusively in the nucleus and functioned as an inhibitor of cell cycle but not as an antiapoptotic protein. In contrast, p27 was constitutively expressed, localized predominantly in the cytoplasm and was not involved in arrest of proliferation. Our data using IκBα mutant LN-18 cells and PI3K/Akt inhibitor-LY294002 revealed that the expression of p21 is regulated by NF-κB. Loss of IκBα function in LN-229 cells (p53 positive) did not influence TNF-α induced accumulation of pp53 (Ser-20 p53) suggesting that p53 was not down stream of NF-κB. Spheroidogenesis enhanced p27 expression and p21 induced by TNF-α was significantly increased in the MCS compared to monolayers.

**Conclusion:**

This study demarcates the functional roles for CDKIs-p21^cip/waf1 ^and p27^kip1 ^during TNF-α stimulated responses in LN-18 glioma cells. Our findings provide evidence that TNF-α-induced p21 might be regulated by NF-κB or p53 independently. p21 functions as an inhibitor of cell proliferation and does not have a direct role in rendering the cells resistant to TNF-α mediated cytotoxicity.

## Background

Gliomas are the most common malignant brain tumors in adults [1]. The median survival of the patients is less than two years and the prognosis remains poor despite extensive research and advances in radiation therapy and chemotherapeutic regimes. The factors responsible for the aggressive behavior of gliomas include marked local invasive growth, neovascularization and evasion to immune responses [2]. Macrophages comprise an important component of the immune system against tumors. In gliomas, macrophages are recruited and remain at the site of tumor constituting a major proportion of the tumor mass [3]. The ability of gliomas to escape the host immune system is thought to contribute to the malignant behavior of these tumors, raising speculation about the role of these tumor infiltrating macrophages in progression and aggressiveness of gliomas [3]. Secretion of cytokines, particularly TNF-α, by activated macrophages is known to affect survival, growth and proliferation of the tumor [4]. TNF-α mediates apoptotic or necrotic effects in many tumors depending on cell types, and causes growth inhibition in 40% of tumor cell lines [5-7]. Reports on the effects of TNF-α on glioma growth and progression are contradictory. TNF-α-induced responses range from cytotoxicity to cytostatic effects [8-10]. Earlier in *vivo *studies with tumors indicated that TNF-α was a death inducer and hence used in clinical trials for treatment of cancers [11]. However recent reports provide evidence that endogenous TNF-α promotes growth and development of tumors [12,13]. In gliomas, TNF-α is shown to reduce growth and prolong survival by enhancing macrophage recruitment and microcyst formation [14]. Understanding the mechanism(s) of TNF-α mediated growth arrest will be important in unraveling the contribution of tissue associated macrophages in tumor resistance in gliomas.

The progression of cells through cell cycle depends on the activation of cyclins and cyclin dependent kinases – CDKs which function together in the G1 phase for initiating S and progression to G2/M phases. The activity of the complexes is regulated by two families of cyclin dependent kinase inhibitors (CDKIs)-including INK4 proteins that bind only to CDK4/CDK6 and are specific for G1 phase and the cip/kip proteins which include p21^cip/waf1 ^and p27 ^kip1 ^and are not specific for any particular cell cycle phase [15,16]. p21 is an universal inhibitor of cell cycle progression and arrests in G1 as well as G2 phases [17]. p21 negatively regulates cell cycle progression by inhibiting CDK2 and CDK4 and blocks DNA replication by binding to PCNA [18]. p21 is also implicated in terminal differentiation, replicative senescence and protection from p53 dependent and p53-independent apoptosis [19,20]. p27 arrests at G1 phase of the cell cycle by contact inhibition and controls the G1/S transition by inhibiting the activity of a wide variety of cyclin/CDK complexes [21]. It is expressed in many tumors and may be partly responsible for growth arrest in tumor cells [22,23]. Though mutations in p27 are rare, a negative correlation between p27 expression and tumor progression is documented in many cancers and loss of p27 in tumors is associated with poor prognosis [24].

Elucidation of cellular and molecular mechanisms involved in survival/death signaling in response to chemotherapeutic agents has been a focus of recent studies for understanding drug resistance in tumors. However, such studies have been hampered by limitations in the conventional *in vitro *models as they do not reflect the complexities of solid tumors. Multicellular spheroids (MCS) closely resemble the *in vivo *situation with regard to cell shape and environment, which in turn can affect the gene expression and behavior of the cells [25]. The biological significance and clinical relevance of MCS have been well documented and these experimental systems have been used for studying micro-environmental effects on basic mechanisms, such as regulation of proliferation, metabolism, differentiation, cell death, invasion, angiogenesis and immune response [26-28]. Though the MCS show greater resistance compared to the two dimensional-monolayer cultures, the mechanisms that confer greater resistance to radiotherapy, chemotherapy in the MCS are not well understood [29]. In this study, we analyzed the effect of TNF-α on proliferation and survival of human glioma cells and investigated the role of CDKIs-p21^cip/waf1 ^and p27^kip1 ^in context with p53 and activation of NF-κB and Akt pathways using monolayer cultures and MCS as *in vitro *models.

## Results

### TNF-α-induced inhibition of proliferation is mediated by TNFR1

**Figure 1:**
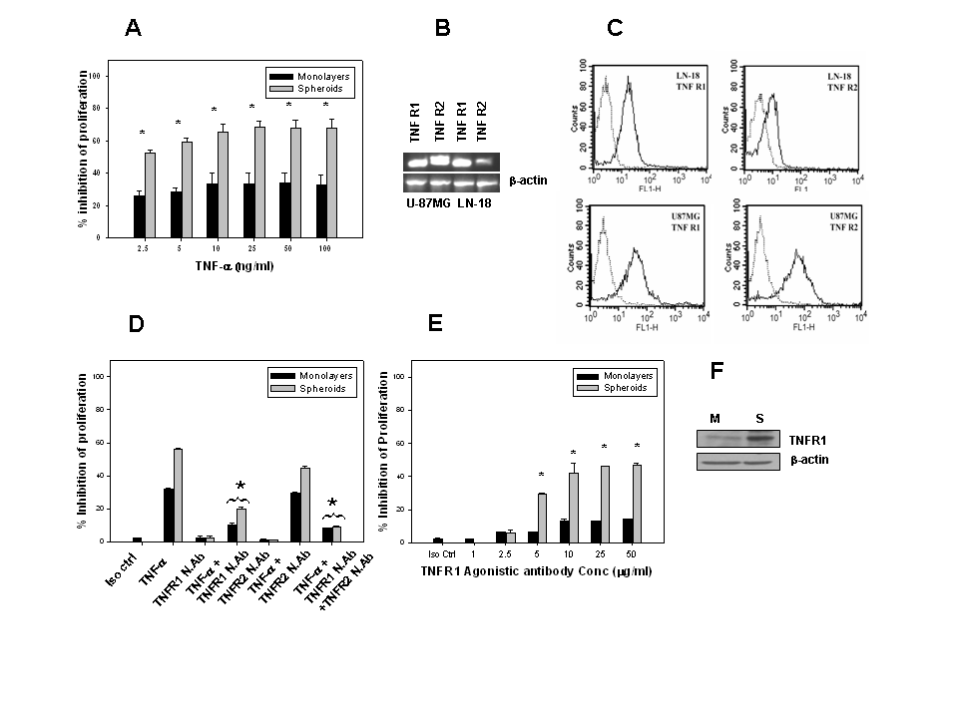
**A-F. TNF-α-induced inhibition of proliferation in LN-18 cells is mediated by TNFR1**. (A) LN-18 cells cultured as monolayers and spheroids for 24 hr were treated with serial concentrations of TNF-α for 24 hr, during the last 18 hr of treatment, 1 μCi of triatiated thymidine was added to the wells. The cells were harvested and the radio activity-counts per minute (CPM) was measured and percent inhibition of proliferation was calculated considering the CPM in untreated cells as 100%. B) Expression of TNFR1 and TNFR2 was analyzed by RT-PCR in LN-18 cells and compared with human glioma cell line-U87MG, used as positive control. β-actin was used as control. C) Expression of TNFR1 and TNFR2 in LN-18 cells and U87MG cells analyzed by flowcytometry, bold lines represent TNFR1/TNFR2 expression and dotted lines depict profiles with isocontrol antibody. D) Monolayers and spheroids were preincubated with neutralizing antibodies to TNFR1 and TNFR2 (50 μg/ml) or isocontrol antibody for 2 hr and treated with TNF-α (10 ng/ml) for 24 hr and proliferation assay was done as described above. E) Monolayers and spheroids were treated with serial concentrations of TNFR1 agonistic antibody or isocontrol antibody for 24 hr and proliferation was determined by triatiated thymidine incorporation assay. The data for A, D and E is the mean ± SE of at least three independent experiments done in triplicates. * p < 0.05-comparison of inhibition of proliferation in TNF-α treated cells in the presence and absence of TNFR neutralizing antibodies for D and between treated and controls cells for A and E. F) Levels of TNFR1 expression by Western blotting in monolayers (M) and spheroids (S) cultured for 24 hr. The blots were stripped and reprobed with β-actin, which served as a loading control of proteins.

Initial experiments were done to assess the effect of TNF-α on cell growth in LN-18 cells. Monolayers and spheroids derived from LN-18 cells were treated with TNF-α and cell proliferation was measured by tritiated thymidine incorporation assay. TNF-α (2.5–100 ng/ml) induced inhibition of proliferation (25%) even at the lowest concentration, with at least two fold greater inhibition (65%) in spheroids at concentrations ≥10 ng/ml (Fig. 1A). TNF-α-induced signaling are mediated via distinct cell surface receptors, TNFR1 (~55 kDa) and TNFR2 (~75 kDa) [30]. The expression of these receptors in LN-18 cells was studied by RT-PCR and flowcytometry analysis. The RT-PCR data and the histoplots show that LN-18 cells express high levels of TNFR1 compared to TNFR2. This was in contrast with another human glioma cell line-U87MG, which showed high expression of both the receptors (Fig. 1B and 1C). To investigate the receptor mediated signaling, cells were pre-incubated with neutralizing antibodies to TNFR1 and TNFR2 (50 μg/ml) and subsequently treated with TNF-α for 24 hr. Pretreatment with neutralizing antibodies to TNFR1 but not TNFR2 reversed the inhibition significantly (Fig. 1D), suggesting that TNF-α stimulated responses were mediated by TNFR1. TNFR1 agonistic antibody (1–50 μg/ml) induced inhibition of proliferation up to 50% at concentrations ≥10 μg/ml in the MCS, while the inhibition was only 15% in the monolayers (Fig. 1E). As the inhibition with TNF-α and TNFR1 agonistic antibody was significantly higher in the spheroids, we determined the levels of TNFR1 in the two culture models by Western blotting and found that the expression of TNFR1 was many fold higher in the spheroids (Fig. 1F). These experiments demonstrated that TNF-α-induced inhibition of proliferation was significantly higher in spheroids compared to monolayers in LN-18 cells and the effect was mediated by TNFR1.

### TNF-α up regulates p21 and p27 expression

**Figure 2:**
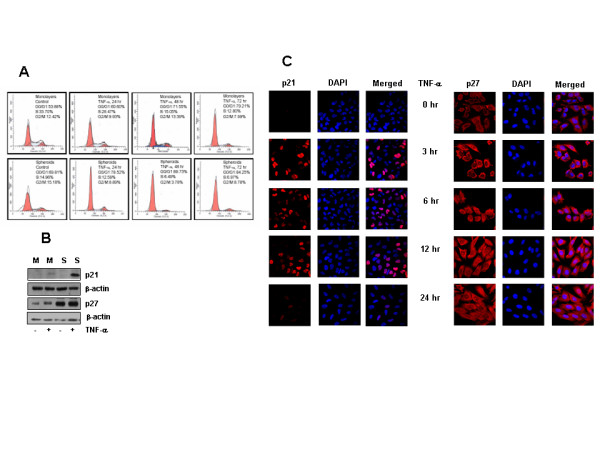
**A-B. TNF-α induced arrest of cells in G0/G1 phase and enhanced expression of p21 and p27**. A) Monolayers and spheroids were treated with TNF-α (10 ng/ml) for 24, 48, and 72 hr and cell cycle analysis was done by flowcytometry. The percent population of cells in G0/G1, S and G2/M phases of cell cycle was determined using Modfit software. The plot is a representative of three similar experiments. B) Monolayers (M) and spheroids(S) were treated with TNF-α (10 ng/ml) for 3 hr and the expression of p21 and p27 was determined by Western blotting. Blots were reprobed for β-actin and used as control for equal loading of proteins. **C. Differential expression and localization of p21 and p27 in LN-18 cells stimulated with TNF-α**. LN-18 cells were grown on coverslips for 24 hr and treated with TNF-α (10 ng/ml) for different time periods. Cells were stained with antibody recognizing nuclear and cytoplasmic p21, or with p27 antibody followed by secondary antibody labeled with Cy3. The expression of p21/p27 is depicted as red fluorescence. DAPI was used for nuclear staining (blue). The merged images show nuclear localization of p21 and p27 localized predominantly in the cytoplasm. (Magnification 63×).

The inhibition in cell growth induced by TNF-α was studied by cell cycle analysis. LN-18 cells were treated with TNF-α up to 72 hr period and analyzed for DNA content by flow-cytometry at 24 hr intervals. Spheroids cultured for 24 hr had higher population of G0/G1 cells compared to the corresponding monolayer. TNF-α treatment significantly increased the cell population in G0/G1 cells in both the models at all the time points but to a greater extent in spheroids compared to monolayer (Fig. 2A)

Cyclin dependent kinase inhibitors (CDKI) p21^cip/waf1 ^and p27 ^kip1 ^are important in TNF-α mediated signaling [31]. We studied the effect of TNF-α on the expression of these CDKI proteins in the two culture models of LN-18 cells by immunoblotting. As shown in Figure 2B, the expression of p21 was below the level of detection in the both the culture systems. On stimulation with TNF-α, the level in monolayers was marginally increased while the expression was strongly enhanced in the spheroids. Our data also revealed that LN-18 cells constitutively expressed p27 and the levels increased drastically with spheroidogenesis. Stimulation with TNF-α resulted in further upregulation of p27 expression in both the models (Fig. 2B). We further performed time course experiments by Confocal Laser scanning Microscopy (CLSM) to study the expression and localization of p21 and p27 in response to TNF-α. We found that p21 was not detectable in the control cells confirming the data from immunoblotting experiments. p21 was induced from 3 hr post-treatment with TNF-α with further increase in the number of p21 positive cells at 6 hr. The level decreased at 12 hr and complete loss of expression was observed at 24 hr. Using an antibody that recognizes p21 in the nucleus as well as cytoplasm it was found that p21 was restricted to the nucleus (Fig. 2C). The cells were highly positive for p27 (>95%) with predominantly cytoplasmic staining though nuclear staining was also seen in some cells. TNF-α treatment marginally enhanced the p27 expression from 3 hr onwards (Fig. 2C).

### TNF-α-induced p21 is regulated by NF-κB and p53 independently

**Figure 3:**
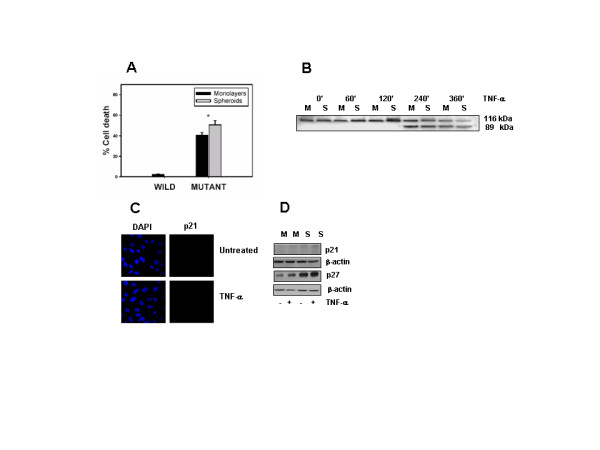
**A-D. Effect of TNF-α on cell viability and expression of p21 and p27 in IκBα mutant LN-18 cells**. (A) Wild and mutant IκBα LN-18 cells were treated with TNF-α (10 ng/ml) for 12 hr and cell viability was assessed by MTT assay. The percent cell death was determined considering viability of control cells as 100%. (B) IκBα mutant LN-18 cells were treated with TNF-α (10 ng/ml) for different time points and cleavage of PARP (89 kD fragment) was determined by Western blotting. C) IκBα mutant LN-18 cells grown on coverslips were treated with TNF-α (10 ng/ml) for 3 hr and stained with p21 followed by secondary antibody labeled with Cy3 and DAPI was used for staining nucleus (Magnification 63×). D) Expression of p21 and p27 determined by Western blotting in monolayers (M) and spheroids (S) derived from IκBα mutant LN-18 cells treated with TNF-α for 3 hr. The blots were stripped and reprobed with β-actin, which served as control for equal loading of proteins.

NF-κB pathway is a one of the major mediators in TNF-α induced effects. As our preliminary experiments indicated that p21 was induced on stimulation with TNF-α in LN-18 cells, we examined the role of NF-κB pathway in the regulation of p21 using a stable IκBα mutant LN-18 cell line. Characterization of the IκBα mutant cell line confirmed that the cells were sensitive to TNF-α mediated cell death. Figure 3A and 3B shows the response of the IκBα mutant cell line to TNF-α determined by MTT assay and PARP cleavage. Western blotting and immunofluorescence analysis revealed that p21 was not detected in the IκBα mutant LN-18 cells and was not induced on stimulation with TNF-α (Fig. 3C, and 3D) suggesting that p21 expression might be regulated by the NF-κB pathway. On the other hand, the expression of p27 was not altered in IκBα mutant LN-18 cells (Fig. 3D and Fig. 2B), implying that p27 expression was not under the control of NF-κB.

**Figure 4:**
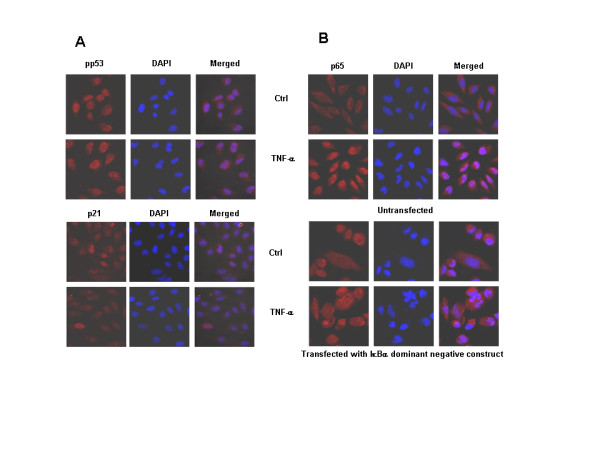
**A-B. Effect of TNF-α on expression of pp53 and p65 in LN-229 cells (p53 positive)**. A) LN-229 cells were exposed to TNF-α for 6 hr and stained with antibodies to phosphorylated Ser-20 p53 and p21. B) LN-229 cells were transfected with IκBα dominant negative construct and the expression of nuclear p65 was determined after exposure to TNF-α for 6 hr.

**Figure 5:**
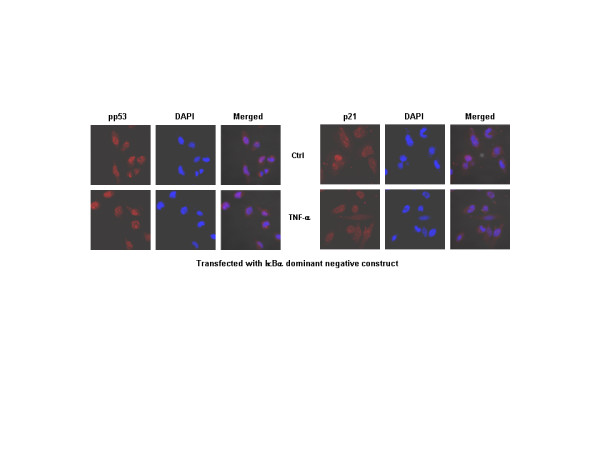
Effect of TNF-α on pp53 and p21 levels was studied in LN-229 cells transfected with IκBα dominant negative construct by confocal laser scanning microscopy. Treatment with primary antibodies was followed by incubation with appropriate secondary antibodies tagged to Cy3. DAPI was used for staining nucleus (Magnification 63×).

p21 is regulated mainly via tumor suppressor protein-p53. Recently, p52/p100 NF-κB was described to regulate p53 function and hence influence the p53-regulated decision-making following DNA damage, a process involving p21 [32]. As LN-18 cells express mutant p53 protein, to examine if NF-κB influenced the p21 expression directly or whether it was via p53, experiments were performed using LN-229, a human glioma cell line expressing wild-type p53 [33]. Cells were exposed to TNF-α for 6 hr and analyzed for the expression of phosphorylated ser20-p53 (pp53) and p21. Stimulation with TNF-α led to enhanced accumulation of phosphorylated p53 in the nucleus, interestingly, the untreated control cells also displayed detectable levels of pp53. In contrast, the expression of nuclear p21 decreased in TNF-α treated cells compared to controls (Fig. 4A). To delineate the pathways regulating p21, the effect of TNF-α on pp53 and p21 was studied in LN-229 cells transfected with IκBα dominant negative construct. The loss of IκBα activity in the transfected cells was evident by the inhibition of p65 translocation to nucleus on stimulation with TNF-α compared with untransfected LN-229 cells (Fig. 4B). To address the question whether loss of NF-κB activation in these cells might influence the levels of pp53 and p21, transfected cells exposed to TNF-α for 6 hr were stained with antibodies to Ser20-p53 and p21. As shown in figure 5, there was no significant difference in the accumulation of pp53 or nuclear p21 between the transfected and untransfected cells. These findings led us to conclude that p53 might not function downstream of NF-κB and secondly, TNF-α-induced p21 can be regulated by p53 and NF-κB independently.

### p21 siRNA did not induce cell death in LN-18 cell line

**Figure 6:**
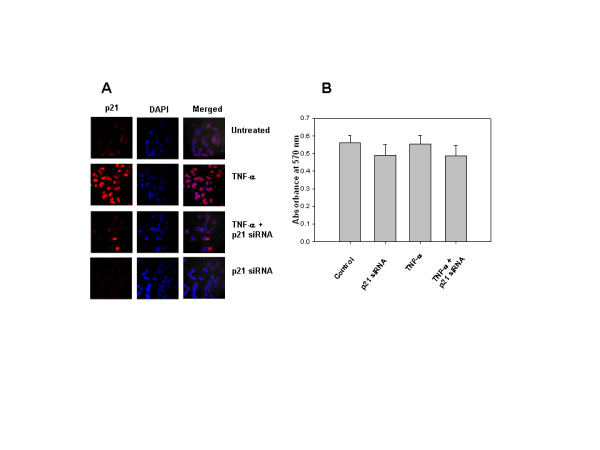
**A-B. TNF-α-induced p21 does not have antiapoptotic function**. (A) LN-18 cells were grown on coverslips and transfected with p21 siRNA oligonucleotides (100 nM) for 24 hr. Control and transfected cells were treated with TNF-α (10 ng/ml) for 3 hr. Cells were stained with p21 antibody followed by secondary antibody labeled with Cy3. DAPI was used for nuclear staining (blue fluorescence), merged images show nuclear localization of p21 (Magnification 63×). (B) Effect of TNF-α on viability in p21 siRNA transfected cells done by MTT assay. The graph represents mean +/- SD of 3 experiments done in duplicates.

Though the role of p21 in cell cycle regulation and growth is well established [19], recently, p21 was reported to also have anti-apoptotic function [34]. The observations that (1) TNF-α induced p21 expression in LN-18 parental cells but not in IκBα mutant cells and (2) IκBα mutant cells were sensitive while the parental cells were resistant to TNF-α mediated cell death prompted us to examine whether p21 functioned as anti-apoptotic molecule in the LN-18 parental cells. For this purpose, cells transfected with p21 siRNA (100 nM) oligonucleotides were treated with TNF-α (10 ng/ml) for 24 hr. The effectiveness of transfection with p21 siRNA was confirmed by determining the expression of p21 by Confocal Laser Scanning Microscopy in transfected cells (Fig. 6A). Results from MTT assay revealed no difference in the viability between the untransfected and transfected cells treated with TNF-α, indicating that p21 did not have a protective role in resistance to TNF-α induced cell death in LN-18 cells (Fig. 6B). The results were confirmed by trypan blue dye exclusion method (data not shown). p21 has to be translocated to cytoplasm and phosphorylated to function as an antiapoptotic protein. In our experiments, as mentioned earlier, p21 was localized exclusively in the nucleus suggesting that p21 was not phosphorylated (Fig. 2C). This observation was confirmed using a phospho-specific p21 antibody (data not shown). These observations suggest that TNF-α-induced p21 in LN-18 cells does not function as an antiapoptotic protein.

### p21 but not p27 was involved in the inhibition of proliferation

**Figure 7:**
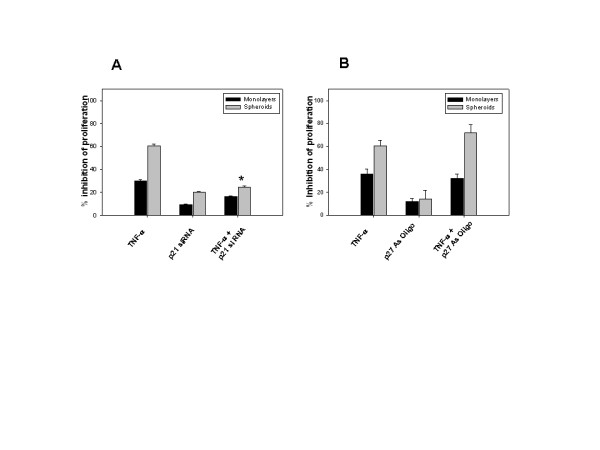
**A-B. p21 but not p27 is involved in TNF-α induced inhibition of proliferation**. (A) LN-18 cells were transfected with p21 siRNA oligonucleotides (100 nM) or (B) p27 antisense (As) oligonucleotides (200 nM) for 6 hr and cultured as monolayers or spheroids for 18 hr. Cells were treated with TNF-α (10 ng/ml) for 24 hr, and tritiated thymidine incorporation assay was done as described in Fig. 1. * p < 0.05 inhibition of proliferation with TNF-α in transfected versus untransfected cells. The data is representative of three experiments done in triplicates.

Further work was done to investigate the role of p21 and p27 in the inhibition of proliferation induced by TNF-α. To this end, cells were transfected with p21 siRNA oligonucleotides and treated with TNF-α (10 ng/ml) and proliferation was assessed by tritiated thymidine incorporation assay. The inhibition was reversed significantly by p21 siRNA oligonucleotides indicating that p21 was involved in arresting the proliferation induced by TNF-α (Fig. 7A). Similar experiments done with LN-18 cells transfected with p27 antisense oligonucleotides (200 nM) revealed no difference in the inhibition between controls and transfected cells treated with TNF-α (Fig. 7B). Together, these results show that p21 did not function as an antiapoptotic protein and secondly p21 but not p27 played a crucial role in the arrest of proliferation induced by TNF-α.

**Figure 8:**
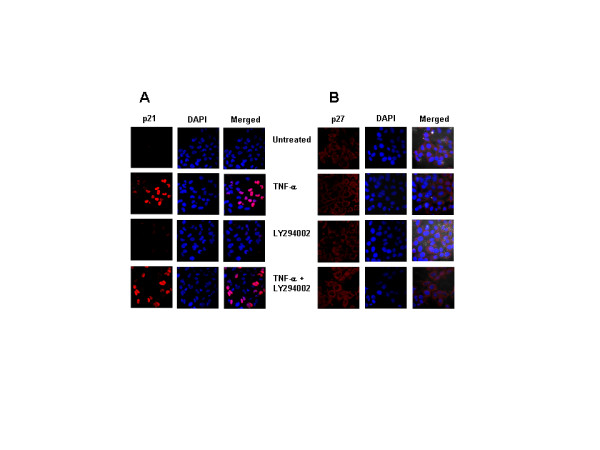
**A-B. Induction of p21 and p27 expression by TNF-α does not involve PI3K/Akt pathway**. (A) LN-18 cells grown on coverslips, were sensitized with LY294002 (50 μM) for 2 hr and treated with TNF-α (10 ng/ml) for 3 hr and the expression of p21 or (B) p27 was studied by confocal laser scanning microscopy. Cells were stained with p21/p27 antibodies followed by secondary antibody labeled with Cy3. DAPI was used for nuclear staining (blue). Merged images depict nuclear localization for p21 and predominant cytoplasmic localization of p27. (Magnification 63×).

The CDKIs, p21 and p27 are reported to be also regulated by PI3K/Akt pathway [35-38], we therefore examined the role of PI3K/Akt pathway by treating LN-18 cells sensitized with LY294002, a pharmacological inhibitor of PI3K/Akt pathway with TNF-α. The inhibitor had no effect on the fluorescence intensity or the percent positivity of p21 or p27 positive cells in TNF-α stimulated cells, suggesting that PI3K/Akt pathway was not involved in regulation of these CDKIs in LN-18 cells (Fig. 8A and 8B).

## Discussion

In this report, we established that though p21^cip/waf1 ^and p27^kip1 ^are upregulated in response to TNF-α, p21 but not p27 is involved in the arrest of proliferation in TNF-α stimulated LN-18 glioma cells. TNF-α-induced inhibition of proliferation was mediated via TNFR1. This observation is significant as most aggressive gliomas express high levels of TNFR1. We also demonstrated that p21 and p27 differed in their localization and expression levels in the experimental models represented by monolayers and spheroids. While p27 was constitutively present, p21 was induced on stimulation with TNF-α and the upregulation in the spheroids was markedly higher compared to monolayers.

p27 is expressed abundantly in human malignant gliomas and is associated with better prognosis [39,40]. p27 is normally a nuclear protein but cytoplasmic localization has been demonstrated in breast and colon cancers and correlates with poor survival in adenocarcinoma [23]. Relocalization of p27 in the cytoplasm is the result of phosphorylation of p27 nuclear localization domain induced by Akt kinase [36] and the presence of cytoplasmic p27 correlates with activated Akt in breast tumors [41]. It is likely that such a correlation exists in gliomas also as high basal levels and activated Akt were detected in LN-18 cells (our unpublished data) and the cells were highly positive for p27 with predominant cytoplasmic localization. Interestingly, PI3K/Akt inhibitor had no effect on the TNF-α-induced p27 upregulation implying that unlike the nuclear p27, cytoplasmic protein may not be regulated via PI3K/Akt pathway. Our findings also rule out the possibility of the involvement NF-κB in regulation of p27 as there was no difference in the expression levels between the parent and the IκBα mutant LN-18 cell lines, which essentially differed in the NF-κB activity. It is believed that p27 translocated to the cytoplasm initiates its degradation and activates CDK2 and can enhance import of cyclin D to the nucleus [41] resulting in the loss of functionality of p27 in cytoplasm. On these lines, it is reasonable to conjecture that the antiproliferative effect of p27 may be functionally impaired in many cancers by sequestration in cyclinD-CDK4/6 complexes and mislocalization in the cytoplasm away from the nuclear targets [16,42]. LN-18 cells were highly positive for p27 and the expression was significantly enhanced on spheroidogenesis. This finding is consistent with the reports that expression of p27 is regulated by contact inhibition and increased level of p27 has been reported in tumor cell lines maintained as spheroids as opposed to cell cultured as monolayers [43]. Though p27 expression was enhanced remarkably on stimulation with TNF-α, experiments with p27-antisense oligonucleotides indicated that p27 did not contribute to the inhibition of proliferation, suggesting the loss of functionality of p27 as an inhibitor of cell cycle. However, the role of cytoplasmic p27 during spheroidogenesis and in response to TNF-α stimulation remains unclear.

Elevated levels of p21 is reported in aggressive gliomas and is used as a prognostic marker [44], surprisingly, p21 was not detectable in LN-18 cells. Evidence for the antiapoptotic function of p21 has been provided in TNF-α induced cytotoxicity in Ewing tumor and MCF-7 breast carcinoma cell line [45], in differentiation induced apoptosis of monocytes [46] and CD95 mediated cell death in human glioma cell lines [47]. In our study, p21 siRNA did not induce cell death in TNF-α-stimulated LN-18 cells, indicating that p21 did not have an antiapoptotic function.

Activation of NF-κB during TNF-α stimulation is believed to one of the major factors contributing to resistance in most tumors. It is speculated that constitutive activation of NF-κB in glioblastoma multiforme (GBM) may be associated with tumor resistance to TNF-α immunotherapy [48]. To investigate the role of p21 in resistance to TNF-α mediated cytotoxicity, we used a stable IκBα mutant LN-18 cell line that was rendered sensitive to TNF-α induced death by transfection with a double mutant construct pcDNA3-IκBα in which both serines (32 and 36) at the amino-terminal are replaced with alanine that are essential for phosphorylation of IκBα during NF-κB activation [49]. In contrast to the parental LN-18 cells, which showed upregulation of p21 in response to TNF-α treatment, p21 was not detectable in the IκBα mutant cells suggesting an association between p21 expression and NF-κB activity. This observation is in accordance with the report demonstrating a correlation between p21 expression and NF-κB activity in Ewing tumor cells [45]. However, other studies demonstrate that p21 induction in daunomycin treated breast and colon carcinoma cell lines does not involve NF-κB though the p21 promoter harbors p53 responsive elements and a functional NF-κB binding site in these cells [50].

In light of a recent report by Schumm *et al*, demonstrating the critical role of p52/p100 NF-κB subunit in p53-dependent p21 regulation in U-2 0S cells [32], it was of interest to investigate the role of p53 in TNF-α-induced p21 in glioma cells. As LN-18 cells have mutant p53, we used another human glioma cell line – LN-229, expressing wild type p53 protein. The tumor suppressor p53 is post-translationally activated by different mechanisms including phosphorylation on serine residues within the C-terminus or N-terminal regions [51,52]. Phosphorylation of p53 at ser20 is an important event in modulation of p53 stability and activity in response to UV and irradiation [53] and is shown to activate p21 in response to DNA damaging agents by enhancing transactivating function of p53 [54]. In this study, we found that TNF-α-enhanced accumulation of pp53 was associated with down regulation of p21 expression in LN-229 cells. Upregulation of p53 accompanied by decreased p21 expression is a typical feature of inhibition of transcription and has been demonstrated in LNCap cells treated with flavopiridol [55]. Negative expression of p21 and overexpression of p53 together is associated with aggressive behavior in gastric tumors [56]. Furthermore, the data from IκBα dominant negative construct transfected LN-229 cells exposed to TNF-α suggested that p53 might not be downstream of NF-κB and p21 could be regulated independently by the p53. Our findings differ from the study with MCF-7 cells reporting that p21 induced on stimulation with TNF-α is not regulated by p53 but by NF-κB [57]. It is note worthy that in LN-229 cells, dexamethosone does not induce p53 expression or alter camptothecin induced p53 expression implying that while regulation of p21 by p53 independent pathway in glioma cells is believed to cell type specific [33], the response may vary also depending on stimuli. Another important finding of this study is that, p53 expression in LN-229 cells was not influenced by the absence of functional IκBα substantiating the conclusion that p53 is not regulated by NF-κB. This observation is differs from the report that IκBα inhibits p53 by binding to it, resulting in cytoplasm p53 and hence prevents nuclear translocation [58].

Recent studies suggest an antiapoptotic function for p21 [45]. To exert protective effect against damage induced by apoptotic agents, it is essential that p21 is phosphorylated and is localized in the cytoplasm [20]. We found that TNF-α-induced p21 was restricted to the nucleus suggesting that it might not function as an antiapoptotic protein. Our conclusion that TNF-α-induced p21 was not phosphorylated and was restricted to nuclear localization is based on our findings using two different antibodies to examine the phosphorylation status of p21, an antibody that was immunoreactive with nuclear as well as cytoplasmic p21 and a second antibody recognizing exclusively phosphorylated p21 (details in materials and methods). Though the molecular mechanisms in compartmentalization of p21 are not clear, it is reported that Akt might regulate the subcellular changes [37]. Phosphotidylinositol 3-kinase (PI3K) and its downstream target, the Akt/PKB serine threonine kinase [59] are activated on TNF-TNFR binding. Akt activity is elevated in most GBM cells particularly those with mutant form of PTEN and is important in glioma formation and progression [60]. Activation of Akt is paralleled with increased p21 stability and correlated with resistance to taxol-induced apoptosis in glioblastoma cell lines [38]. High grade gliomas are known to express high levels of Akt [52] and enhanced Akt activity is demonstrated in spheroids from Ewing tumors [53]. We observed increased levels and activity of Akt in spheroids of LN-18 cells (our unpublished data). These findings and our data depicting increased expression of TNF-α-induced p21 in spheroids compared to monolayer cells prompted us to examine the involvement of Akt in the regulation of p21. We found that PI3K/Akt inhibitor-LY290042 did not affect the expression of p21 suggesting that it was not regulated by this pathway. Experiments with p21 siRNA reversed the inhibition of proliferation confirming its role as a regulator of cell cycle progression. These results led us to conclude that p21 functioned as an inhibitor of proliferation but not as an antiapoptotic protein in TNF-α mediated response in LN-18 cells. Furthermore, our studies with IκBα mutant LN-18 cells and pharmacological inhibitor-LY290042 revealed that p21 expression was regulated by NF-κB and not Akt pathway.

In conclusion, this study demarcates the functions of CDKIs-p21^cip/waf1 ^and p27^kip1 ^during TNF-α stimulated responses in LN-18 glioma cells. p21 induced on stimulation with TNF-α may be regulated by NF-κB or p53, probably depending on the status of p53. Our findings do not support a role for p27 in regulation of proliferation or rendering resistance in TNF-α induced cytotoxicity, which could be attributed to its cytoplasmic localization. However questions concerning the relevance of enhanced cytoplasmic p27 expression during spheroidogenesis and in response to TNF-α stimulation remain to be answered.

The findings in this study assume importance from the point of TNF-α-TNFR1 interaction in the *in vivo *milieu in gliomas wherein TNF-α is secreted by the tumor infiltrating macrophages and TNF receptors are abundantly expressed by the tumor cells. The stimulation leads to activation of various pathways including NF-κB pathway which contributes to chemoresistance. The resistance may be further enhanced by induction of p21 by TNF-α in NF-κB dependent manner leading to the arrest of cells in the G1 phase. The regulation of p21 by p53 and NF-κB in wild type p53 and mutant p53 tumors in response to TNF-α deserves further in-depth studies Additionally, heightened induction of p21 to TNF-α stimulation in MCS representing the cells in low proliferating phase underscore the MCS as more realistic model for studying the molecular mechanisms in resistance to chemotherapeutic agents in solid tumors.

## Methods

### Cell cultures and generation of multicellular spheroids

The human glioma cell lines LN-18, LN-229 and U87MG were procured from American Type Culture Collection (ATCC, U.S.A). Cells were cultured in Dulbecco' s modified eagle's medium (DMEM) supplemented with 1.5 gm/L sodium bicarbonate, 4 mM glutamine and 5%-10% fetal bovine serum (Gibco, U.S.A) at 37°C in an atmosphere of 5% CO2/95% air. Multicellular spheroids were generated by the liquid overlay technique as described previously [61] with a slight modification. All experiments were done using 24 hr cultures. Single cell suspensions were prepared from spheroids by dissociation with 0.25% trypsin in PBS at 37°C for 30 min.

### Cell viability assay

The effect of TNF-α on cell viability was assessed by MTT (3-(4, 5-Dimethyl-2-thiazolyl)-2, 5-diphenyl-2H-tetrazollium bromide) assay. Cells were treated with TNF-α (1–100 ng/ml) for 24 hr. During the last 4 hr of incubation, 10 μl of MTT (5 mg/ml) (Amersham Biosciences, U.S.A) were added. The crystals were dissolved in 10%SDS-0.01N HCl and absorbance was measured at 570 nm with reference to 650 nm. The percent viability was calculated considering controls as 100%. Cell death was also confirmed by trypan blue dye exclusion method.

### Tritiated thymidine incorporation assay

Cell proliferation was measured by tritiated thymidine incorporation assay. Cells were grown as monolayers or spheroids in 96 well plates and treated with serial concentrations of TNF-α (1 to 100 ng/ml – Peprotech, U.S.A) for 24 hr, during the last 18 hr before termination of the assay, 1 μCi tritiated thymidine (Specific activity 240 Gbq/mmole, from Board of Radiation and Isotope Technology-BRIT, India) were added to wells. Monolayer cells were trypsinized in the same plate while spheroids were transferred to fresh plate and harvested using cell harvester (Nunc, Denmark). The radio activity-Counts per Minute (CPM) were measured using a liquid scintillation counter (Packard, U.S.A). The percent inhibition was calculated, considering incorporation in control (untreated) cells as 100%.

### Cell cycle analysis

Cells were plated at 2 × 10^6^/100 mm dish in duplicates and treated with TNF-α (10 ng/ml) up to 72 hr. Cells were harvested at 24 hr interval post-treatment, stained with propidium iodide (50 μg/ml) and DNA content was determined on the FL-2A channel with flowcytometer equipped with 488 nm argon laser (FACS Vantage – BD Sciences, U.S.A). The data was analyzed using Modfit software to determine population in different phases of cell cycle.

### Western blotting

Cells were lysed in RIPA buffer (50 mM Tris-HCl, pH 7.4, 150 mM NaCl, 1% TritonX-100, 1% NP-40, 1% Sodium deoxycholate, 0.1% Sodium dodecyl sulphate, 2 mM Phenylmethylsulfonylfluoride, 50 mM Sodium fluoride, 5 mM iodoacetamide, 2 mM β-glycero-phosphate, 1 mM benzamidine, 1 mM sodium orthovanadate) and protease inhibitor cocktail (Roche, Germany). Protein samples were separated by SDS-PAGE (10–15%) and electro-transferred onto PVDF membrane (Amersham BioSciences, U.S.A). The expression of proteins was analyzed using antibodies to p21 and p27 (Cell Signaling and Santacruz Biotechnology, U.S.A), TNFR1 (R&D Systems, U.S.A). The blots were incubated with appropriate secondary antibodies conjugated to horse radish peroxidase (Biorad, U.S.A), and developed using ECL PLUS kit (Amersham Biosciences, U.S.A). The protein levels were normalized by reprobing the blots with antibody to β-actin (ICN, U.S.A).

### Confocal laser scanning microscopy

Cells grown for 24 hr on cover slips were treated with TNF-α (10 ng/ml) for different time points. Cells were washed twice with PBS, fixed with 3.7% paraformaldehyde for 10 min at RT. Cells were permeabilized with 0.2% Triton-X 100 for 5 min and blocked with PBS containing 1% BSA and 10% goat serum for 30 min. Cells were incubated with appropriately diluted antibodies to pp53, p65, and p27 (SantaCruz Biotechnology, U.S.A) p21, pp21(Cell Signaling Technology, U.S.A) overnight, washed and incubated with corresponding secondary antibodies labeled with Cy-3 for I hr. Cells were washed with PBS, stained with DAPI (0.5 μg/ml) and mounted in mounting media (Oncogene, U.S.A) and viewed under Confocal microscope (Zeiss LSM 510, Germany) equipped with argon laser and helium lasers. The controls comprised of cells processed with isocontrol antibody or without primary antibody. For assessing the role of PI3K/Akt pathway in the regulation of p27 and p21, cells were incubated for 2 hr with LY290042 (Calbiochem, U.S.A), a pharmacological inhibitor of PI3K/Akt prior to TNF-α treatment.

### Cell transfections and generation of IκBα mutant stable LN-18 cell line

IκBα mutant stable cell line was generated using a double mutant construct in which both serines (32 and 36) at the amino-terminal of IκBα, are mutated to alanine. DNA was mixed with 10 μl lipofectamine 2000 (Invitrogen, U.S.A) in 500 μl of serum free medium for 30 min and layered over the LN-18 cells grown at 50% confluence and after 6 hr the medium was changed. After incubation for 24 hr, the cells were grown in selection medium (G418, 600 μg/ml) and clones were picked and plated into 96 well plates. Single cell colonies were grown in G418 (400 μg/ml). Transfections done with empty vectors comprised as controls. The mutant status in the cell line was confirmed by analyzing the response to TNF-α by IκBα degradation and p65 nuclear localization. Transient transfections were done with IκBα dominant negative construct in LN-229 cells.

### Antisense oligonucleotide transfection

LN-18 cells were transfected with 200 nM of p27 phosphorothioate labeled antisense oligonucleotides synthesized by Life technologies, U.S.A. The sequences were as follows – p27 antisense-5'-gacactctcacgtttgacat-3', scrambled-5'-ttgccgctgctaagttcg-3', using oligofectamine (Invitrogen, U.S.A) in serum free medium. After 6 hr, the medium was supplemented with serum, cultured for 24 hr and treated with TNF-α (10 ng/ml). Western blot analysis for confirming p27 expression and tritiated thymidine assays were performed as described above.

### Transfection with p21 siRNA

Cells were cultured for 24 hr and transfected with p21 siRNA oligonucleotides (50 nM) according to the manufacturer's instructions (Cell Signaling Technology, U.S.A). After 24 hr, medium was replaced and cells were treated with TNF-α (10 ng/ml) for 3 hr and processed for p21 staining, proliferation and viability assays as described above.

### Statistical analysis

Statistical analysis was performed using one-way analysis of variance (ANOVA) followed by students *t*-test or Fisher's exact test. A p-value < 0.05 was considered to be statistically significant.

## Authors' contributions

PSK contributed to the major part of experimental work and the data was a part his PhD thesis. GD contributed to the development of stable LN-18-IκBα double mutant cell lines. JCJ and VP helped in flowcytometry and confocal microscopy studies. AS contributed to stimulating discussions and critical comments on the article. PS is the principal investigator, responsible for concept of the project and designing the experiments.
